# The suboptimal health status questionnaire: Turkish validity and reliability study

**DOI:** 10.3389/fpubh.2026.1762572

**Published:** 2026-05-29

**Authors:** Fatma Birgili, Nezihe Bulut Uğurlu, Nevin Güler Dincer, Güllü Yazkan

**Affiliations:** 1 Department of Nursing, Faculty of Health Sciences, Muğla Sıtkı Koçman University, Muğla, Türkiye; 2 Department of Statistics, Faculty of Science, Muğla Sıtkı Koçman University, Muğla, Türkiye; 3 Department of Nursing, Health Sciences Institute, Muğla Sıtkı Koçman University, Muğla, Türkiye

**Keywords:** nurse, reliability, scale, suboptimal health status, validity

## Abstract

**Background:**

Suboptimal health status (SHS), while understood worldwide, is a widespread public health problem and a reversible condition that presents symptoms in the pre-disease process, ranging from optimal health to illness. Therefore, relevant questionnaires need to be developed across diverse populations and cultures to predict and prevent SHS and to inform personalized medical practices. Therefore, this study aims to adapt the Suboptimal Health Status Questionnaire to Turkish and evaluate its validity and reliability among Turkish nurses.

**Methods:**

Between January and March 2022, 247 nurses were recruited by convenience sampling at Muğla Training and Research Hospital. Confirmatory factor analysis (CFA) and reliability analysis were performed, respectively, to test the construct validity and reliability of the scale.

**Results:**

Results indicated excellent reliability (content validity index = 0.78, Cronbach’s α = 0.95). To investigate the time invariance of the scale, the intraclass correlation coefficient (ICC) was used, and ICC values were found between 0.64 and 0.89. CFA results, goodness-of-fit measures were found to be within the limits of perfect fit (root mean square residual = 0.048, goodness-of-fitness index = 0.971, adjusted goodness-of-fitness index = 0.965, normalized goodness-of-fitness index = 0.966, relative goodness-of-fitness index = 0.961, and parsimony normalized goodness-of-fitness index = 0.853). Spearman’s rho coefficient between the first and second applications was found to be 0.76. The correlations between subscale scores obtained from the first and second applications ranged from 0.62 to 0.88. All correlations were positive and statistically significant (*p* < 0.05).

**Conclusion:**

The Turkish version of the SHSQ-25 scale was found to be a reliable and valid measurement instrument and can be used to measure SHS among Turkish nurses.

## 1 Introduction

Nursing is considered one of the most stressful, tiring, and challenging professions, and nurses regularly experience work-related problems such as long working hours, shifts, patient complaints, and low income. Considering the long working hours of nurses in the healthcare field, nursing is known to be a profession requiring commitment and sacrifice. Although nursing is a profession that demands professional dedication and identification, it is inevitable that these individuals will experience occupational stress and burnout due to the heavy workload. Nurses are expected to provide higher-quality healthcare services by adopting a more holistic approach to human beings and dedicating more time to human values, placing human beings at the center of the nursing profession. This will lead to more effective healthcare services, resulting in increased patient satisfaction, employee satisfaction due to stable job opportunities, and more efficient use of human resources through improvements in the workplace (1–3). Nurse burnout is related not only to physical condition but also to emotional and mental health. In this case, nurses’ professional efficiency, productivity, and performance, and consequently the quality of patient care, can be negatively affected. Therefore, paying attention to both the physical and mental health of nurses is crucial.

Health, while a universal concept, is a fundamental inherent right. The World Health Organization (WHO) constitution defines health as: “*Health is not merely the absence of disease and disability, but a state of complete physical, mental and social well-being*.”

In this definition, physical and mental wellbeing are the known aspects of health. “Social well-being” is a new concept requiring further explanation (4).

Suboptimal health status (SHS) is an optimal state of health between health and disease, caused by physical, psychological, and social stress experienced by individuals. While the underlying causes of SHS are not yet fully understood, changes in the endocrine and immune systems can impair physical, mental, and social functioning.

These changes, which are not yet at the disease stage, reduce the body’s coordination. SHS manifests itself with many symptoms such as pain, fatigue, dizziness, depression, loss of appetite, and sleep disturbances. It can also cause negative effects on the digestive, cardiovascular, respiratory, and urinary systems.

SHS can also increase visits to healthcare facilities, which increases healthcare costs and can impair quality of life (5–8). Therefore, SHS is often associated with the onset of chronic diseases.

Risk factors for passive smoke exposure include smoking, alcohol consumption, skipping breakfast, an unbalanced diet, lack of exercise, and sleep problems (8, 9).

Although SHS does not meet the diagnostic criteria for any diagnosable medical condition, it reflects the subclinical and reversible stage of a chronic condition, with the perception of health complaints, general malaise, and low energy (10–13).

Most people with SHS have one or more risk factors other than chronic infectious disease (6). These risk factors include obesity (14), hypertension (15), musculoskeletal disorders (16), environmental factors (17), bad habits that negatively affect health (18), and physical and mental factors (19).

**Figure 1:**
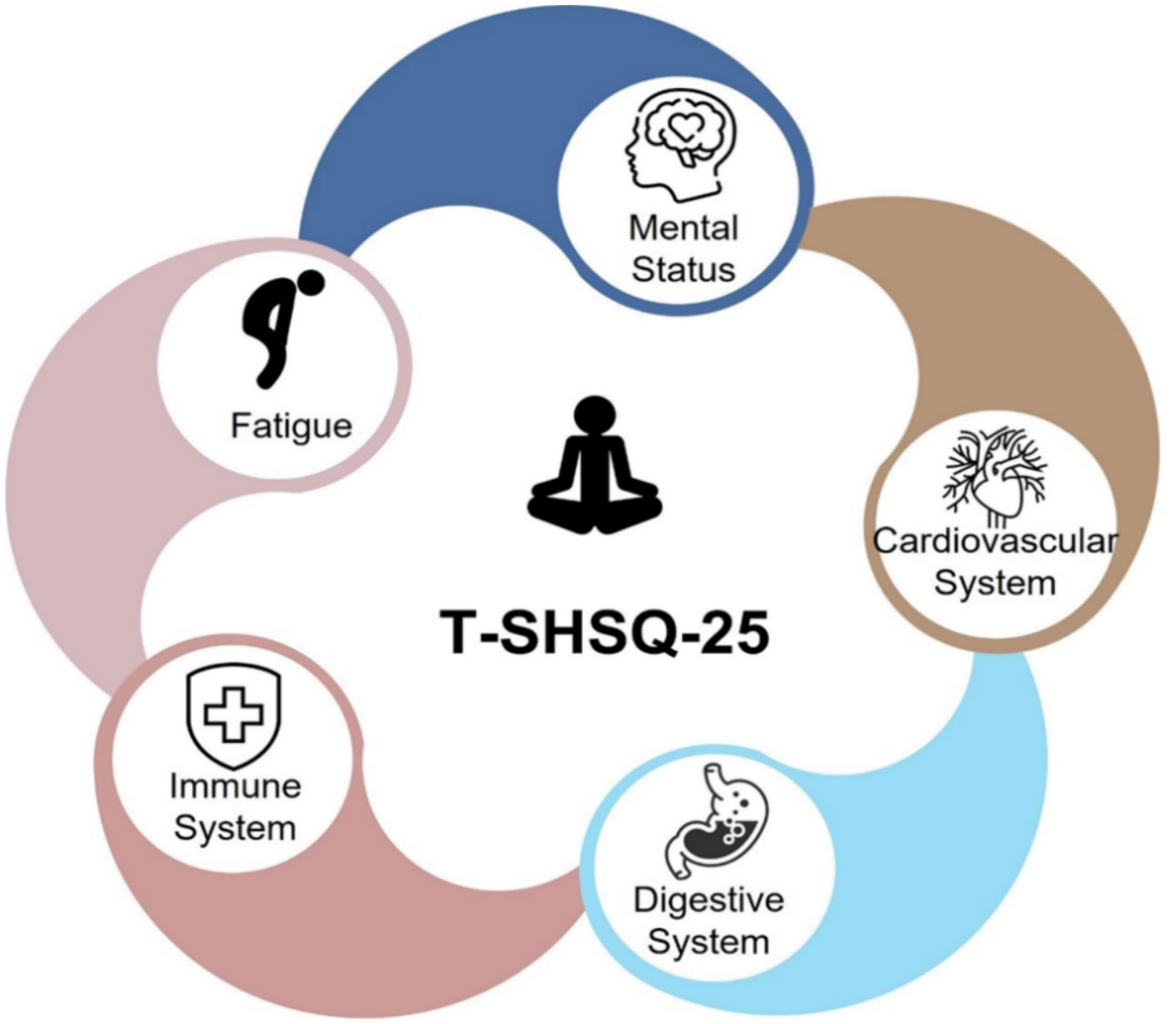
Turkish version of the T-SHSQ-25: five domains.

The concept of SHS has led to the development of the five-dimensional (Figure 1) and 25-item (20) non-invasive self-report questionnaire, the T-SHSQ-25, which measures SHS according to the perspective of predictive, protective, preventive, and individualized medicine.

This questionnaire has been validated in three main ethnic groups: African, Asian, and Caucasian, and has good validity and reliability (3, 9, 12, 21). However, there is currently no Turkish version of this questionnaire available.

This study aimed to translate the SHSQ-25 from English into Turkish linguistically and then cross-culturally examine its validity and reliability in a Turkish population (22, 23).

## 2 Methods

### 2.1 Sample and procedures

This research was conducted using a methodological design to translate the T-SHSQ-25 from English into Turkish and to validate the reliability and validity of the Turkish version for use in a Turkish population.

When adapting a scale to another culture, it is necessary to reach at least 5 to 10 times the number of items in the scale to determine the sample size (22, 23). Therefore, since the T-SHSQ-25 has 25 items, it was aimed to conduct the study with 250 nurses willing to participate, which is 10 times the number of items in the SHSQ-25.

The study universe consisted of 444 nurses working at the Muğla Education and Research Hospital. The aim was to reach 10 times the number of scale items, but the sample consisted of 247 nurses who agreed to participate in the study and could be reached.

Among the 444 nurses working in the hospital, 247 of them (72.5% females) were recruited. The mean age of the males (30.76 ± 6.81 years) was younger than that of the females (32.08 ± 7.27 years).

### 2.2 Measurements

In this study, a questionnaire including the “Socio-Demographic Characteristics Form” and “SHSQ-25” was used to collect research data.

The Personal Information Form included eight questions related to nurses’ education level, daily physical activity level, current job pressure, daily sleep time, smoking and alcohol use, chronic disease, and medicine use, created by researchers in line with the literature (22, 23).

SHSQ-25 has 25 items and 5 dimension: fatigue (9 items: 1–6, 8–10), cardiovascular system (3 items: 11–13), digestive system (3 items: 14–16), immune system (3 items: 7, 17, 25), and mental state (7 items: 18–24).

Each participant answered the 5-point Likert-type questionnaire questions according to the frequency with which they experienced different specific complaints in the last 3 months before starting the study. These were: (0) never or almost never, (1) sometimes, (2) often, (3) very often, and (4) always (20).

In the determination of the validity, the researchers administered the SHSQ-25 to the nurses by face-to-face interviews with the permission of the clinic administration.

Inclusion criteria for participants in the study were: (1) no history of somatic or psychiatric abnormalities, (2) being over 18 years of age, (3) not having used any medication in the last 2 weeks, and (4) not having any disease.

### 2.3 Statistical analysis

Data were coded and evaluated using the statistical package programs SPSS Version 22.0 and Amos 22.0.

Davis’ content validity ratio formula was used for the content validity of the scale.

Confirmatory factor analysis (CFA) was performed to evaluate construct validity. Cronbach’s *α* analysis was used to test internal consistency in the reliability analysis.

Average variance extracted (AVE) and composite reliability (CR) were used to assess convergent validity. CR values greater than 0.7, and AVE values greater than 0.5 were considered acceptable (24).

Squared inter-construct (SIC) was utilized to evaluate the discriminant validity. If the AVE values of the factors are greater than the SIC values, the construct demonstrates discriminant validity (25).

The Wilcoxon signed-rank test was used in the test–retest analysis to determine whether there was a statistically significant difference in the mean scores obtained from administering the questionnaire at two different time points, in terms of the total SHS scale score and its subdimensions scores.

Spearman’s rho coefficient was also used in the test–retest analysis to examine the correlation between the total scores obtained from the two administrations.

The one-sample Kolmogorov–Smirnov test was used to assess whether continuous variables followed a normal distribution.

The Student’s *t*-test was used to compare the means of two independent samples with normal distribution, while one-way analysis of variance (ANOVA) was used to compare the means of more than two groups.

For variables that did not meet the assumption of normality, the Mann–Whitney *U*-test was used for comparisons between two groups, and the Kruskal–Wallis test was used for comparisons among more than two groups.

The statistical significance value was set at a *p*-value of < 0.05 in all tests (22).

### 2.4 Validity study

The validity study of T-SHSQ-25 consists of three main steps: language validity, content validity, and construct validity.

### 2.5 Language validity of the T-SHSQ-25

**Figure 2:**
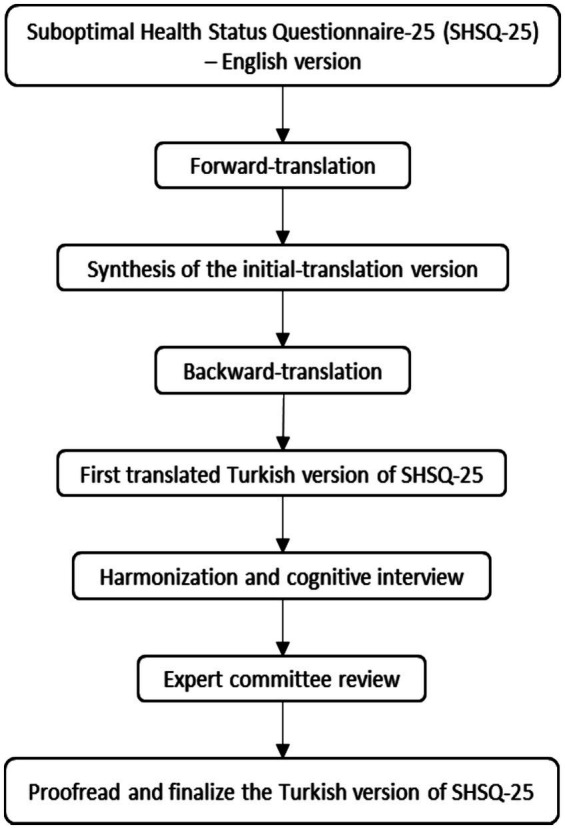
Flow chart of the translation process.

Following the WHO guidelines for translation and adaptation of instruments (26), the SHSQ-25 was translated from English into Turkish using the forward-backward translation method for language validity (Figure 2).

The Turkish translation of the T-SHSQ-25 questionnaire, originally in English, was carried out by five different translators.

The five initial translated versions were evaluated by the researchers and advisors, and the most appropriate expressions were merged into a single draft after achieving consensus.

The expressions in the entries were evaluated by a Turkish linguistics expert in terms of clarity, comprehensibility, and spelling rules.

### 2.6 Content validity of the T-SHSQ-25

When a scale is adapted to a different culture, it is recommended to consult at least three experts to determine the linguistic equivalence of the items in the original and translated forms (27).

In this context, the English and Turkish forms of the scale were reviewed by a group of nine experts.

The experts used a 4-point Likert scale (1 = not at all appropriate, 4 = completely appropriate) to rate each item in terms of comprehensibility, appropriateness, and relevance.

The item content validity index (CVI) was calculated using a formula that subtracts one from the ratio of the number of experts who gave 3 and 4 points to the total number of experts.

The scores obtained were 3–4 points from 7 out of 9 experts (78%), 2 points from 1 out of 9 experts (11%), and 1 point from 1 out of 9 experts (11%).

The CVI was determined by dividing the number of experts who received an acceptable score (3–4) by the total number of test evaluations (28).

Based on the experts’ comments, necessary corrections were made to the questionnaire.

T-SHSQ-25 was then sent back to two Turkish–English bilingual translators for translation to eliminate any potential semantic shifts.

The final version of the T-SHSQ-25 questionnaire was created after correcting spelling, grammar, and formatting errors in the items.

Following expert review, the draft scale was piloted on 16 nurses with similar characteristics to the students to be included in the study; these nurses were not included in the sample.

Since each item was found to be understandable in the pilot study, further analyses were conducted.

### 2.7 Construct validity of the T-SHSQ-25

In evaluating the item and dimensional structure of T-SHSQ-25, CFA, goodness-of-fitness index (GFI), adjusted goodness-of-fitness index (AGFI), normalized goodness-of-fitness index (NFI), relative goodness-of-fitness index (RFI), Parsimony normalized goodness-of-fitness index (PNFI), and root mean square residual (RMR) goodness-of-fit tests were performed (29).

### 2.8 Reliability study

To assess the temporal invariance of the draft scale and its five sub-dimensions, test–retest mean scores were compared using a significance test of the difference between the two means, and the intraclass correlation coefficient (ICC) was calculated.

Accordingly, values below 0.50 indicate low reliability, values between 0.50 and 0.75 indicate moderate reliability, and values above 0.75 indicate good reliability.

Cronbach’s *α* coefficient, ranging from 0 to 1, was used to assess the internal consistency reliability of the T-SHSQ-25 (30, 31).

Cronbach’s *α* values are as follows: “(≥0.9) excellent, (0.8–0.9) good, (0.7–0.8) acceptable, (0.6–0.7) doubtful, (0.5–0.6) weak, and (≤0.5) unacceptable.”

The absence of a difference after repeated measures showed that the scale measured similar results at intervals, indicating consistency between measurements.

### 2.9 Ethical approval

Following the planning process, ethical committee forms for scientific research were prepared, and after obtaining ethical committee approval (protocol number 210105, decision no: 115) and written institutional permission from the Muğla Sıtkı Koçman University Health Sciences Ethics Committee, where the research was conducted, the data collection process began.

Participants were informed of the research’s purpose and benefits on the data collection form, and informed consent was obtained from the nurses, ensuring the principle of voluntariness. Participants completed the forms anonymously.

In the first application, nurses were asked to fill in the code on the data collection form and were instructed to write the same code in the test–retest application. The research was conducted in accordance with the principles of the Helsinki Declaration.

## 3 Results

### 3.1 General characteristics of participants

A total of 247 participants completed the investigation. Most participants were females (*n* = 179; 72.5%), and 68 were males (27.5%). Of these, 85.8% (*n* = 212) were university graduates, 10.9% (*n* = 27) were vocational high school graduates, and 3.2% (*n* = 8) were postgraduates.

The mean age of the participants was 31.72 years (SD = 7.15) (Table 1).

**Table 1:** Characteristics of questionnaire respondents.

Characteristic		Female (*n* = 179)	Male (*n* = 68)
Age (years)		32.08 ± 7.27	30.76 ± 6.81
Sleeping time		6.40 ± 1.52	6.81 ± 1.78
Education	University	153 (85.5%)	59 (86.8%)
	Vocational high school	21 (11.7%)	6 (8.8%)
	High school	0 (0%)	0 (0%)
	Postgraduate	5 (2.8%)	3 (4.4%)
Smoking	Yes	95 (53.1%)	47 (69.1%)
	No	84 (46.9%)	21 (30.9%)
Drinking	Yes	62 (34.6%)	39 (57.4%)
	No	117 (65.4%)	29 (42.6%)
Level of physical activity	Primarily motionless	0 (0%)	0 (0%)
	Sedentary with frequent activity	14 (7.8%)	0 (0%)
	Primarily physical	134 (74.9%)	53 (77.9%)
	High-intensity activity	31 (17.3%)	15 (22.1%)
Current work pressure	Not at all	3 (1.7%)	3 (4.4%)
	A little	29 (16.2%)	15 (22.1%)
	Middle	78 (43.6%)	26 (38.2%)
	High	53 (29.6%)	18 (26.5%)
	Very high	16 (8.9%)	6 (8.8%)
Clinic/unit	Internal units	41 (22.9%)	16 (23.5%)
	Surgical units	43 (24%)	18 (26.5%)
	Emergency/surgery room/intensive care unit	84 (46.9%)	32 (47.1%)
	Management	0 (0%)	1 (1.5%)
	Polyclinic	11 (6.1%)	1 (1.5%)

According to Table 1, the mean age of the female participants was 32.08 ± 7.27, while the mean age of the male participants was 30.76 ± 6.81. The average daily sleep duration was 6.40 ± 1.52 for female participants and 6.81 ± 1.78 for male participants.

There was no statistically significant difference between the mean ages of female and male participants (*p* = 0.201). However, the difference in sleep duration was statistically significant (*p* = 0.021), with male participants having longer sleep duration.

### 3.2 Construct validity findings of T-SHSQ-25

In the study, the factor loadings of each domain in fatigue ranged between 0.677 and 0.859, immune system between 0.400 and 0.520, cardiovascular system between 0.860 and 0.935, digestive tract between 0.907 and 0.920, and mental status between 0.636 and 0.792.

All factor loadings were greater than 0.4. These findings indicated that the multidimensional structure of the instrument generally replicated the conceptualization of the original five domains.

**Figure 3:**
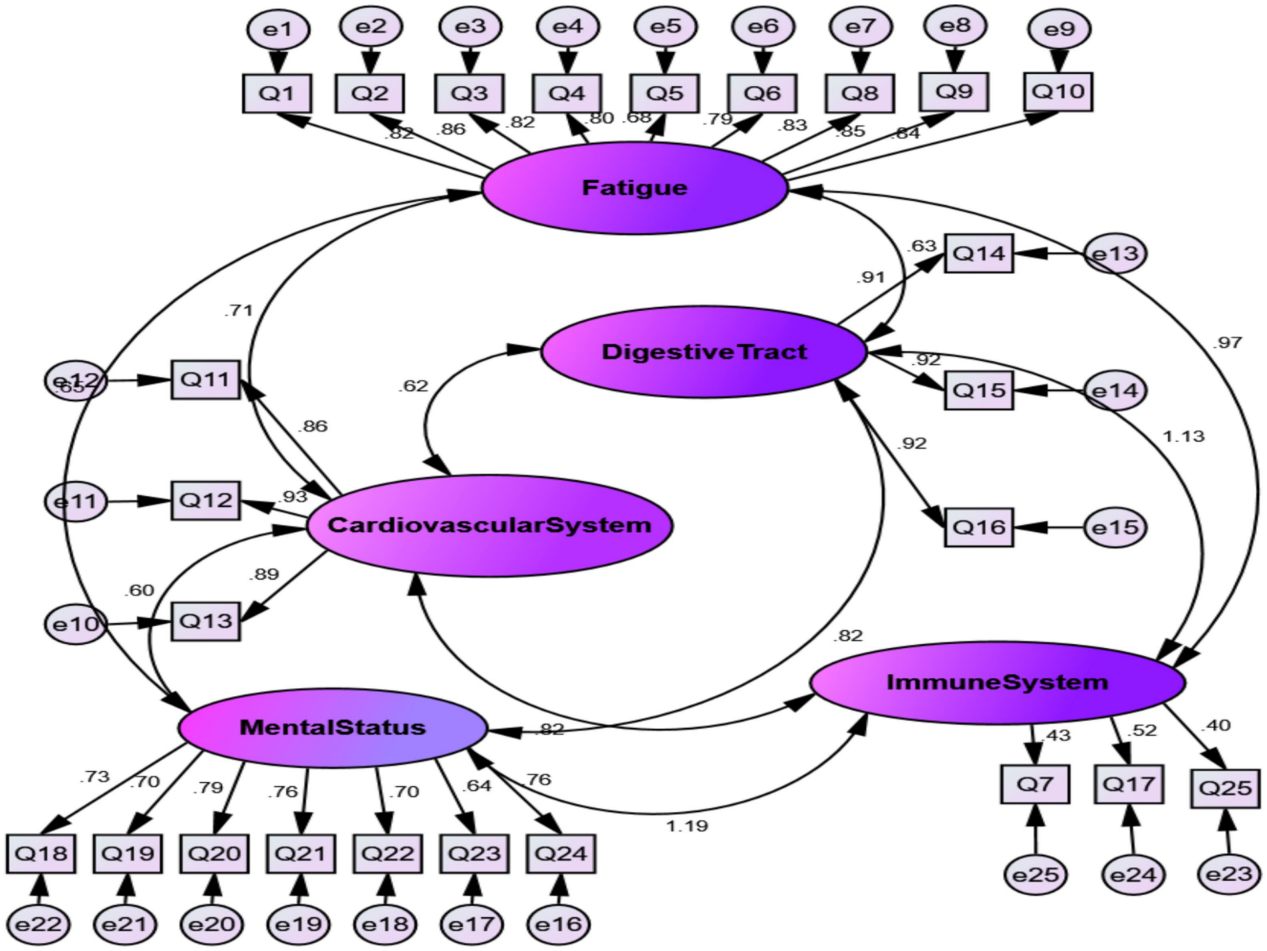
Confirmatory analysis of the 5 dimensions and 25 items of the T-SHSQ-25.

CFA was further performed to confirm the factor structure of the T-SHSQ-25. As results of CFA without modification, the path diagram is shown in Figure 3.

CFA based on five intercorrelated factors showed a reasonable fit of the data to the factor structure: GFI = 0.971 > 0.95 perfect fit, AGFI = 0.965 > 0.95 perfect fit, NFI = 0.966 > 0.95 perfect fit, RFI = 0.961 > 0.95 perfect fit, PNFI = 0.853 > 0.95 perfect fit, > 0.5 acceptable fit, RMR = 0.048 < 0.05 perfect fit, < 0.08 acceptable fit.

Construct validity measures were as follows: GFI, 0.95 ≤ GFI ≤ 1.00 good fit, 0.90 ≤ GFI ≤ 0.95 acceptable fit; AGFI, 0.90 ≤ AGFI ≤ 1.00 good fit, 0.85 ≤ AGFI ≤ 0.90 acceptable fit; NFI, 0.95 ≤ NFI ≤ 1.00 good fit, 0.90 ≤ NFI ≤ 0.95 acceptable fit; RFI, 0.90 < RFI < 1.00 good fit, 0.85 < RFI < 0.90 acceptable fit; PNFI, takes values in the range of 0–1, but higher values indicate more economical fit.

PNFI, takes values ​​in the range of 0–1, but higher values ​​indicate more appropriate fit. RMR takes values in the range of 0–1. As the RMR value approaches 0, it indicates a good fit, whereas higher values indicate a poor fit (25).

It can be seen that the values of all goodness-of-fit measures were within the limits of a perfect fit. Construct reliability (CR) and average variance extracted (AVE) values were found to be 0.945 and 0.657 for fatigue, 0.924 and 0.801 for cardiovascular system, 0.939 and 0.837 for digestive tract, 0.886 and 0.528 for mental status, and 0.433 and 0.205 for immune system, respectively.

These results indicate that the fatigue, cardiovascular system, digestive tract, and mental status constructs show acceptable levels of validity.

However, the immune system construct exhibits low CR and AVE values, indicating that it does not adequately meet the required criteria for convergent validity.

Regarding discriminant validity based on SIC, moderate relationships were observed between fatigue and the cardiovascular system (*r*^2^ = 0.506), the digestive tract (*r*^2^ = 0.394), and mental status (*r*^2^ = 0.419), suggesting adequate discrimination among these constructs.

Similarly, acceptable levels of discriminant validity were observed between the cardiovascular system and the digestive tract (*r*^2^ = 0.382) as well as the mental status (*r*^2^ = 0.365).

However, the immune system construct demonstrated excessively high squared correlations with other constructs, including fatigue (*r*^2^ = 0.937), cardiovascular system (*r*^2^ = 0.667), digestive tract (*r*^2^ = 1.275), and mental status (*r*^2^ = 1.411).

These values indicate a lack of discriminant validity, suggesting that the immune system construct is not sufficiently distinct from the other latent variables. In this study, exploratory factor analysis was not performed since the factor structure of the original SHSQ-25 was confirmed, and it was not necessary to extract the new factors for the Turkish sample (31).

### 3.3 Reliability of the T-SHSQ-25

Table 2 presents test–retest and internal consistency reliability results.

**Table 2:** Test–retest and internal consistency results.

Scale	Application	Mean ± SD	Wilcoxon signed-rank *p*-value	Spearman’s rho correlation	ICC (95% confidence interval)	Cronbach’s *α*
SHS	First	48.77 ± 11.11	0.55	0.76**	0.80** (0.64, 0.89)	0.95
	Second	49.63 ± 10.20				
Fatigue	First	22.17 ± 5.45	0.60	0.68**	0.64** (0.39, 0.80)	0.94
	Second	22.34 ± 5.87				
Immune system	First	5.80 ± 1.45	0.18	0.76**	0.72** (0.51, 0.85)	0.45
	Second	5.60 ± 1.19				
Cardiovascular system	First	4.31 ± 1.55	0.06	0.77**	0.76** (0.57, 0.87)	0.92
	Second	4.66 ± 1.49				
Digestive tract	First	4.34 ± 1.49	0.34	0.88**	0.89** (0.80, 0.94)	0.94
	Second	4.46 ± 1.42				
Mental status	First	12.14 ± 3.13	0.38	0.62**	0.67** (0.44, 0.82)	0.89
	Second	12.57 ± 3.12				

N, number; SD, standard deviation; ICC, intraclass correlation coefficient; CI, confidence interval.

*p < 0.05, **p < 0.01.

ICC values ranged from 0.64 to 0.89 (Table 2). The results showed that there were significant correlations between the first and second measurements. In summary, the Turkish version of SHSQ-25 satisfies the requirement of time invariance.

Except for the domain of the immune system, Cronbach’s α values of other domains in T-SHSQ-25, including fatigue, cardiovascular system, digestive tract, and mental status, were 0.8 or higher, indicating good internal consistency. The Cronbach’s *α* value of the immune system domain indicated moderate reliability since the value was between 0.4 and 0.6 (27).

### 3.4 Comparison of total SHS scale scores by general characteristics

In this section, the results of comparisons of the total SHS scale according to certain characteristics of the participants are presented.

To conduct hypothesis testing, that is, to increase the sample size within groups, some groups in the variables of clinic/unit, level of physical activity, and current work pressure were combined.

Table 3 presents the results of the tests examining whether the mean scores of the total SHS scale differ significantly according to general characteristics.

**Table 3:** Results of the comparison test of total SHS scale score means.

Characteristic	Category	Mean ± SD	*p*
Gender	Female	47.63 ±12 .83	*0.004
	Male	43.06 ±11 .66	
Age	21–30	40.39 ± 7.57	*<0.001
	31–40	49.73 ± 12.07	
	41–50	57.73 ± 15.75	
Educational level	Vocational high school	41.63 ± 7.25	0.119
	University	46.96 ± 13.17	
	Postgraduate	46.75 ± 10.47	
Smoking	Yes	48.27 ± 13.90	*0.020
	No	43.80 ± 10.26	
Drinking	Yes	47.75 ± 13.22	0.208
	No	45.41 ± 12.21	
Drug	Yes	51.92 ± 11.93	0.120
	No	46.09 ± 12.66	
Chronic disease	Yes	52.95 ± 12.69	*0.012
	No	45.76 ± 12.51	
Clinic/unit	Internal units	47.86 ± 13.51	0.221
	Surgical units	47.52 ± 12.80	
	Other	45.16 ± 12.18	
Level of physical activity	Low	39.36 ± 7.55	*0.039
	Middle	46.22 ± 12.65	
	High	49.11 ± 13.23	
Current work pressure	Low	40.48 ± 8.24	**0.000
	Middle	43.67 ± 9.80	
	High	52.55 ± 14.81	
Sleeping	<5	46.00 ± 10.97	0.607
	≥5	46.44 ± 12.99	

*p < 0.05, **p < 0.001.

When Table 3 is examined, the mean total SHS scale scores show statistically significant differences according to gender, age, smoking status, presence of chronic disease, daily physical activity level, and current work pressure characteristics (*p* < 0.05).

In addition, it can be stated that the mean SHSQ scores are higher among individuals who are female, aged 41–50, have a history of smoking, have a chronic disease, and have high levels of daily physical activity and current job pressure.

## 4 Discussion

SHS is not a condition of probability. However, it is a condition that usually appears before clinical symptoms such as pain, fatigue, and insomnia and before the disease is diagnosed. This condition is reversible and plays a very important role in predicting and managing chronicity (32).

SHS helps prevent the occurrence of the condition, supports early detection and intervention, and reduces unnecessary hospital visits and expenses (33).

The current study aimed to provide a Turkish version of the T-SHSQ-25 suitable for suboptimal health research in the Turkish-speaking population.

In accordance with the WHO’s international guidelines for translating health materials across different languages and cultures, a translation and back-translation process was carried out. After translation, the content validity analysis was conducted based on the established six stages, including forming the expert group, preparing the candidate questionnaire forms, getting expert opinions, obtaining the content validity rates for the items, reaching the content validity index of the questionnaire, and creating the final form according to the content validity rates (28).

The CVI obtained in the current study, which was used to estimate the quality of content validity, indicated that the language of the T-SHSQ-25 was understandable and the contents were appropriate.

The results of the current pilot survey demonstrated that the T-SHSQ-25 is a reliable, robust, and valid tool to assess the SHS in Turkish nurses.

The test–retest analysis demonstrated adequate reliability (ICC = 0.80, 95% CI: 0.64–0.89) and internal reliability (Cronbach’s *α* = 0.95) that corresponded to values observed with the English version of SHSQ-25 (ICC = 0.93; 95% CI: 0.91–0.95; Cronbach’s *α* = 0.93) (20), and the Korean version of SHSQ-25 (ICC = 0.95; 95% CI: 0.88–0.99; Cronbach’s *α* = 0.95) (34).

To test the test–retest reliability of the questionnaire, the Turkish forms were investigated in 35 nurses with 2-week intervals, and the correlation coefficient between the two measurements was 0.76 (*p* < 0.001).

A statistically positive and high-level correlation was found between the first and second total questionnaire scores in the test–retest analysis. Based on these findings, it could be concluded that the test–retest reliability coefficient of the T-SHSQ-25 was sufficient.

To examine the construct validity, we performed CFA on the five domains of the T-SHSQ-25. The factor loadings of the items ranged from 0.40 to 0.935. According to the goodness-of-fit criteria of CFA, the results revealed a good fit of the data based on the GFI (0.971), AGFI (0.965), NFI (0.966), RFI (0.961), PNFI (0.853), and RMR (0.048), and all the criteria indexes reached the respective standards.

Although the overall model fit indices indicated a good fit to the data, the immune system construct showed relatively lower factor loadings compared to other dimensions. Overall, these findings suggest that while most constructs demonstrate satisfactory psychometric properties, the immune system construct fails to meet validity criteria. This may indicate that the items related to this construct were interpreted differently in the target cultural context, and that cultural differences may have negatively affected its psychometric performance in the adaptation process. Because the concept of SHS was derived from traditional Chinese medicine and Turkey is a Middle Eastern country, cultural differences between countries and regions should be considered to ensure that the questionnaire is adapted to the local culture and maintains linguistic equivalence to the original version during the process of translation (20, 31).

Our findings demonstrated that the T-SHSQ-25 encompassed 5 domains and 25 items, which are consistent with the English version of the SHSQ-25.

The results showed that there was no difference in the factor pattern between the Turkish and English versions of SHSQ-25, and the successfully cross-culturally translated T-SHSQ-25 can be utilized as a valid and reliable instrument to assess SHS and describe the health properties in Turkish society (20).

Although we achieved the idiomatic and validated T-SHSQ-25, there are limitations in our study that need to be addressed. This study was conducted in a single center, and participants were limited to the nurse population. Therefore, future studies should be conducted in a larger sample and validated in a multicenter replication.

Nursing is considered one of the most stressful and challenging professions. Occupational stress is a common complaint among nurses (35). Common workplace stressors faced by nurses include long working hours, work schedules, patient complaints, and low salaries (1). In addition to physical side effects such as pain, fatigue, and weakened immune systems, this demanding job can seriously affect mental health and quality of life (34). SHS generally manifests as impairment in physiological, emotional, and social functioning (20, 34). The decision to identify SHS is made if the individual has experienced symptoms without an underlying disease state for the previous 3 months (36). In this study, significant differences were found in the total SHSQ-25 scores according to gender, age, smoking status, presence of chronic disease, daily physical activity level, and current work pressure characteristics. In one study, the prevalence of SHS among nurses in hospitals during the 2019 coronavirus disease (COVID-19) pandemic was reported as 35.1% (37). In another study, it was observed that more than half of the nurses (54.5%) experienced SHS. This situation shows that nurses are under serious pressure due to complex working conditions in the hospital and poses a threat to the physical and mental health of nurses (35). Accordingly, SHS can affect the physical and mental health of nurses, patient safety, and the quality of hospital and healthcare services. Therefore, it is important to assess the SHS situation of nurses and take necessary measures.

## 5 Conclusion

In conclusion, the Turkish version of the SHSQ-25 had a sufficient level of agreement with the original questionnaire with five domains. The Turkish version of the T-SHSQ-25 questionnaire was found to be equivalent to the English version in terms of language, culture, and concepts. Adding to the English, Chinese, Russian, and Korean versions of SHSQ-25, the current study generated an applicable T-HSHQ-25. The questionnaire is easy to understand, accept, and apply to evaluate the health conditions of the Turkish-speaking population. Further, we recommend the T-SHSQ-25 as a screening tool for early detection of chronic diseases and for helping to reduce the burden of diseases.

## Data Availability

The raw data supporting the conclusions of this article will be made available by the authors, without undue reservation.
